# Ovarian Vein Embolization With N-butyl-2 Cyanoacrylate Glubran-2^®^ for the Treatment of Pelvic Venous Disorder

**DOI:** 10.3389/fsurg.2021.760600

**Published:** 2021-12-16

**Authors:** Maofeng Gong, Xu He, Boxiang Zhao, Jie Kong, Jianping Gu, Haobo Su

**Affiliations:** Department of Interventional and Vascular Radiology, Nanjing First Hospital, Nanjing Medical University, Nanjing, China

**Keywords:** pelvic venous disorder, chronic pelvic pain, Glubran-2, ovarian vein embolization, NBCA (N-butyl-2- cyanoacrylate)

## Abstract

**Background:** There are few reports in the literature on the use of Glubran-2 for the embolization of ovarian veins in patients with pelvic venous disorder (PeVD). In addition, a consensus on the efficacy and safety of Glubran-2 has not been reached.

**Purpose:** To investigate the safety and efficacy of ovarian vein embolization (OVE) with N-butyl-2 cyanoacrylate (NBCA) Glubran-2 for the treatment of PeVD.

**Material and Methods:** Between January 2013 and January 2020, 21 women (mean age, 43.9 ± 13.3 years) with PeVD who underwent OVE with Glubran-2 were evaluated. Of those patients, ovarian vein or pelvic venous plexus insufficiency was verified by duplex ultrasound and/or multislice computer tomography (MSCT). The symptoms and signs of PeVD included chronic pelvic pain (CPP) (21/21; 100%), dyspareunia (12/21; 57.1%), dysmenorrhea (10/21; 47.6%), and vulvar varices (3/21; 14.3%). The medical data were retrospectively reviewed.

**Results:** Glubran-2 was employed as the sole embolic material in 18 cases (85.7%) and used to perform rescue embolization in 3 cases (14.3%) due to CPP recurrence 1 month after initial embolization using microcoils. Technically successful embolization was achieved using Glubran-2 in all patients. No Glubran-2 related complications were noted. Neither persistent nor recurrent CPP was observed during follow-up, for which the mean was 62 ± 38 months (range, 12–102 months). Clinical efficacy was evaluated, and all patients exhibited complete or slight improvement of CPP after embolization. The visual analog scale (VAS) score significantly decreased from pre-intervention to post-intervention (*p* < 0.001). Six patients (28.6%) gave birth to healthy babies during follow-up after embolization with Glubran-2.

**Conclusions:** Ovarian vein embolization with Glubran-2 is a feasible and safe treatment for CPP secondary to PeVD. This treatment may represent a potential and attractive alternative when patients desire symptom relief and want to continue reproducing. Larger studies are warranted to confirm the findings of this study.

## Introduction

Chronic pelvic pain (CPP) is one of the most severe symptoms of the pelvic venous disorder (PeVD) caused by valvular incompetence of ovarian veins and pelvic venous plexuses (1). Chronic positional pain varies in terms of intensity and duration and is frequently accompanied by dyspareunia and urgent bladder irritability, which can extend to the posteromedial thigh or buttock and has an extremely negative impact on quality of life (QOL) (2, 3). Despite increasing awareness of this condition, the precise etiology of CPP secondary to PeVD remains uncertain and is probably related to multiple factors. Pelvic vein incompetence with the retrograde flow in the varicose utero-ovarian plexus has been implicated as one of the most important causes of CPP secondary to PeVD (4).

Ovarian vein embolization (OVE) has been recommended in cases with a 2B level of evidence for the treatment of PeVD, has a low rate of morbidity or complications, and has largely replaced open surgical intervention (5, 6). The goal of OVE is to occlude incompetent varicose veins to reduce excessive blood flow within the pelvic vein plexus. As previously reported (7, 8), conventional embolic materials used for OVE include sclerosing agents and metal coils and have a clinical efficacy ranging from 58 to 93%. However, sclerotherapy involves a difficult monitoring process using fluoroscopy, and coils pose risks of migration, hypersensitivity, or further recanalization (7–13). Although, a consensus on which embolic materials are most suitable for OVE is lacking.

Liquid embolic materials, such as N-butyl-2 cyanoacrylate (NBCA), are efficiently used to achieve hemostasis to treat acute arterial hemorrhage in various organs (14). In particular, the considerable advantage of a high rate of polymerization makes NBCA a potential alternative in PeVD. Nevertheless, to the best of our knowledge, there are few reports in the literature on the embolization of PeVD with Glubran-2 for the treatment of CPP, and consensus on the efficacy and safety of this treatment has not been reached. The purpose of the present study was to investigate the safety and efficacy of OVE with NBCA Glubran-2 for the treatment of PeVD.

## Methods

### Study Population

**Figure 1:**
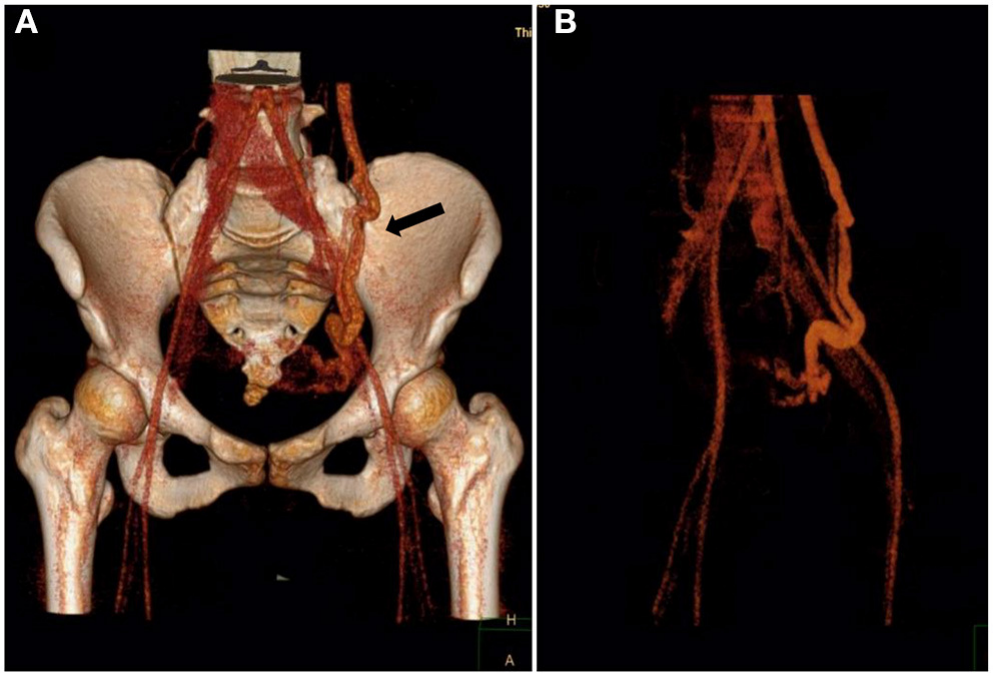
Three-dimensional reconstruction of contrast-enhanced multislice computer tomography (MSCT) revealed varicose gonadal veins (black arrows). The diameter of the vein was 8 mm, and deposition of contrast agent was noted in the pelvic veins. **(A)** Anteroposterior. **(B)** Loxosis.

This retrospective study was approved by our institutional review board, and written consent was obtained from the patients involved. A search of medical databases between January 2013 and January 2020 was performed, and 18 patients who had contracted CPP secondary to PeVD without pelvic outflow obstruction and underwent OVE with Glubran-2 as the primary embolic material were selected. In addition, 3 patients who were treated using microcoils and Glubran-2 as a rescue therapy were included. The mean female patient age was 43.9 ± 13.3 years, and the CPP duration was 27.8 ± 11.6 months. Glubran-2 was employed in 21 patients, including 18 who underwent left unilateral OVE and 3 who underwent right unilateral OVE. Of the included cases, OVE with Glubran-2 was performed to treat symptoms of CPP (21/21; 100%), dyspareunia (12/21; 57.1%), dysmenorrhea (10/21; 47.6%), and vulvar varices (3/21; 14.3%) (Table 1). All patients underwent transvaginal duplex ultrasound to exclude endometriosis or adenomyosis, and 9 patients were confirmed to have PeVD from an additional contrast-enhanced MSCT (Figure 1) performed before pelvic venography.

**Table 1:** Demographics, presentation, and lesion characteristics of patients with chronic pelvic pain (CPP) who underwent ovarian vein embolization with N-butyl-2 cyanoacrylate (NBCA) Glubran-2.

**Characteristic**	**Value**
Age, *y*, mean ± SD (range)	43.9 ± 13.3 (25–65)
Female sex, *n* (%)	21 (100)
Duration of symptoms at presentation, months, mean ± SD (range)	27.8 ± 11.6
Parity, mean (range)	2.5 (1–6)
Gravidity, mean (range)	1.3 (0–3)
**Location**, ***n*** **(%)**	
LOV	18 (85.7)
ROV	3 (14.3)
**Medication**, ***n*** **(%)**	
Tramadol	3 (14.3)
Aspirin	4 (19.0)
Ibuprofen	2 (9.5)
**Clinical presentation**, ***n*** **(%)**	
CPP	21 (100)
Dyspareunia	12 (57.1)
Dysmenorrhea	10 (47.6)
Vulvar varices	3 (14.3)
**CPP level (VAS score)**, ***n*** **(%)**	
No pain	0 (0)
Mild pain	3 (14.3)
Moderate pain	6 (28.6)
Severe pain	12 (57.1)

*CPP, chronic pelvic pain; LOV, left ovarian vein; ROV, right ovarian vein; VAS, visual analog scale*.

*Continuous data are presented as a mean ± SD; categorical data are presented as counts (percentages)*.

### N-butyl-2 Cyanoacrylate Embolization Procedure

The embolic material used in the present study was Glubran-2^®^ (N-butyl-2 cyanoacrylate; GEM, Viareggio, Italy). The benefits and potential risks of embolization with Glubran-2 were explained to patients and/or their relatives, and detailed informed consent was obtained in all cases. The therapeutic approach was left to the discretion of the treatment group, which consisted of 2 interventional radiologists with at least 15 years of experience each.

A 4-French (F) sheath (Radifocus Introducer II Introducer Sheath; Terumo, Leuven, Belgium) was initially inserted under local anesthesia into the femoral vein. To confirm the diagnosis of the varicose ovarian vein and uterine vein engorgement, overview venography was initially performed before embolization using a 4-F Cobra catheter (Radifocus Angiographic Catheter; Terumo, Leuven, Belgium) with and without the Valsalva maneuver. When incompetent segments of ovarian and uterine veins were identified, a compatible 2.4 F microcatheter (Progreat; Terumo, Leuven, Belgium) was subsequently coaxially positioned, ensuring that the microcatheter tip was as close as possible to the target varicose veins of the utero-ovarian plexus. The dead space of the microcatheter was initially loaded with a 5% dextrose solution to prevent early polymerization of Glubran-2 in the lumen of the microcatheter. To facilitate visualization, Glubran-2 was thoroughly mixed with ethiodized oil (an ethiodized poppy seed oil injection was obtained from Hengrui Medicine, Jiangsu, China; the concentration ratio of Glubran-2 to oil ranged from 1:3 to 1:4, depending on a subjective evaluation of the distance from the microcatheter tip to the vein site and the vein flow speed). Under fluoroscopy control, the mixture was injected as slowly as possible using thumb pressure, which was adjusted according to the rate of Glubran-2 propagation in the varicose veins and target vein flow speed. The Glubran-2 injection was continued until the varicose veins were completely occluded to prevent undesired embolization of normal vein branches to the maximum extent possible. At the end of Glubran-2 embolization, the microcatheter was removed rapidly, and microcoils (MWCE; COOK, Bjaeverskov, Denmark) were employed for 4 patients at the opening of the varicose ovarian vein trunk to reduce the risk of non-target embolization from Glubran-2 overflow. Final venography was performed through the Cobra catheter to demonstrate vein occlusion.

### Definitions of Efficacy, Safety, and Follow-Up

The efficacy of OVE with Glubran-2 was evaluated both technically and clinically (15). Technical success was defined as complete occlusion of the target incompetent varicose ovarian vein and reflux pelvic veins on the final venography. CPP was quantified during pre-intervention, post-intervention, and follow-up on a scale of 0–10 using the visual analog scale (VAS) and classified into four categories: no pain (0–1), mild pain (2–4), moderate pain (5–7), and severe pain (8–10) (16). There were three categories of clinical symptom improvement: complete, corresponding to a decrease in the VAS score to 0–1; slight, corresponding to a reduction in the level of pain by one or two categories; and no improvement, corresponding to the VAS score remaining in the same category or worsened. Clinical success was defined as a complete or slight improvement in the clinical symptoms of CPP without the need for repeat endovascular treatment or surgery. Safety was evaluated based on complications noted during both mid-Glubran-2 embolization and post-intervention, and the occurrence of adverse events mid-Glubran-2 injection was especially noted. Clinical efficacy based on the VAS score was evaluated during follow-up by telephone for all patients at the 1st, 3rd, and 6th months and 6-month intervals thereafter. For any instance of an increased VAS category of CPP, a consultation for a clinical examination (by transvaginal Doppler ultrasound or enhanced CT) was offered. A questionnaire on QOL, which included questions on symptoms (CPP worsened with standing/sitting/walking; dysmenorrhea; dyspareunia; and presence of varices), was conducted at the 12th month. The degree of improvement in patients' symptoms after embolization was scored as follows: 0, no complaints and the complete absence of discomfort in daily life; 1, clear improvement (indication of only mild remaining symptoms that were not disabling); 2, moderate improvement (indication of remaining symptoms, which although fewer in number than before the procedure was performed, could be disabling); 3, no improvement; and 4, worsening symptoms.

### Statistical Analyses

The SPSS statistical software package (version 23.0; SPSS statistical software, Chicago, Illinois, USA) was used to perform all statistical analyses in this study. Continuous variables are expressed as the mean±standard deviation. Qualitative variables are presented as percentages. The correlation between pre-intervention and post-intervention variables was assessed using a paired *t*-test. Findings with a *p* < 0.05 were deemed statistically significant.

## Results

**Figure 2:**
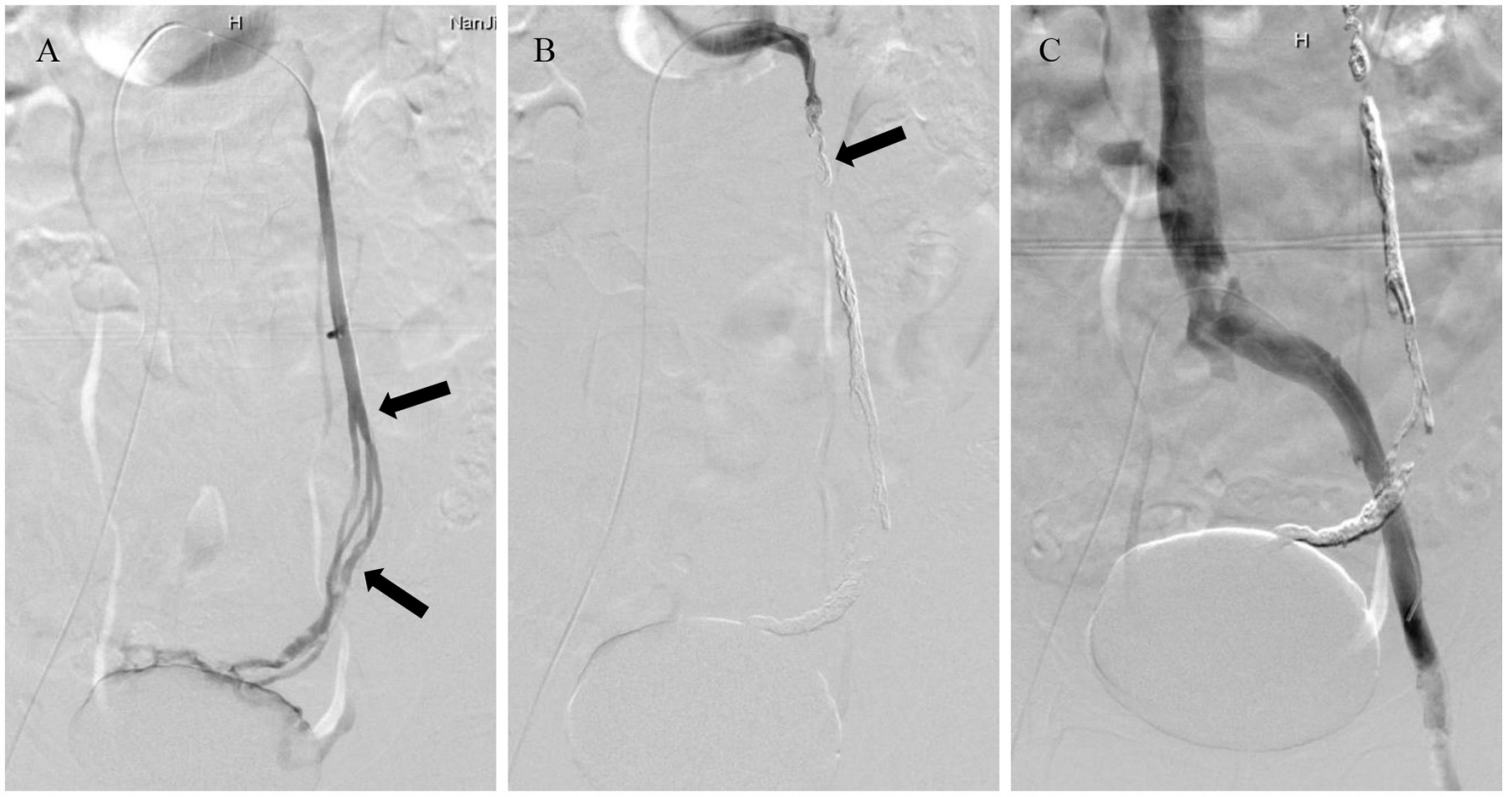
A 59-year-old woman with chronic pelvic pain secondary to pelvic venous disorder. **(A)** Selective left ovarian vein venography demonstrated retrograde flow in a dilated incompetent gonadal vein (black arrows). **(B)** Ovarian vein embolization with Glubran-2. Specifically, the varicose gonadal veins were completely occluded, and the opening of the ovarian vein trunk was sealed with microcoils (black arrow). **(C)** Final venography of the left iliac vein after embolization revealed no reflux flow draining into the utero-ovarian plexus.

**Figure 3:**
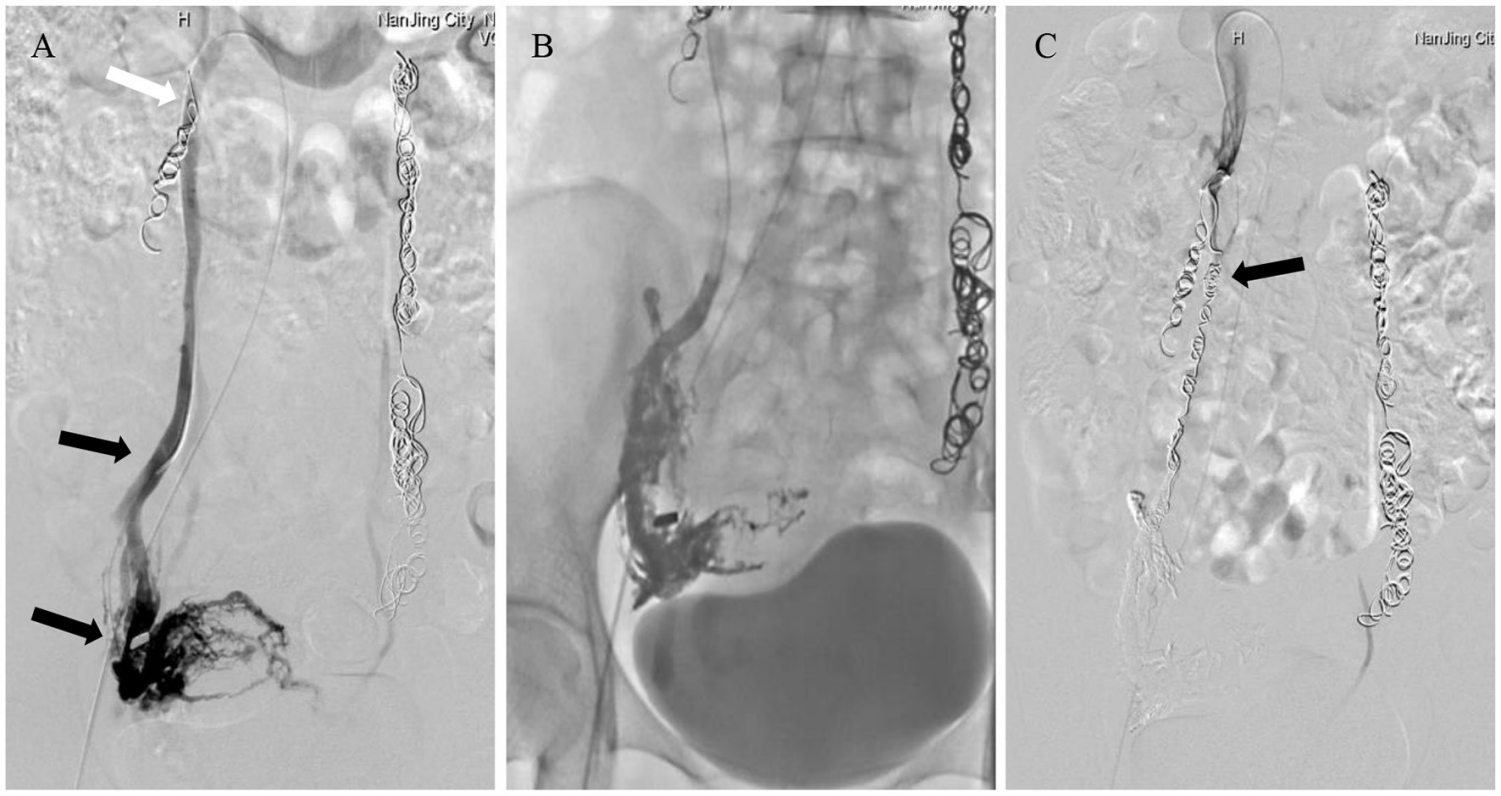
A 38-year-old woman underwent a second ovarian vein embolization (OVE) with Glubran-2 due to the recurrence of chronic pelvic pain 1 month after an initial embolization using microcoils. **(A)** Right ovarian vein venography demonstrated reflux flow in a dilated varicose ovarian vein and uterine vein engorgement extending across the midline (black arrows). The ovarian vein consisted of multiple trunks, and the tails of the microcoils that were initially used were partially inserted into the main trunk (white arrow). **(B)** OVE with Glubran-2, where the varicose gonadal veins were completely occluded. **(C)** The opening of the ovarian vein trunk was sealed with microcoils (black arrow), and no retrograde flow was observed during right ovarian vein post-embolization with Glubran-2 and sealing with microcoils.

Glubran-2 was employed as the sole embolic material in 18 cases. In 4 cases, Glubran-2 embolization was augmented using microcoils at the opening of the varicose ovarian vein trunk to reduce the risk of non-target embolization from overflow during the procedure (Figure 2), and the mean number of microcoils used was 4 ± 1. Glubran-2 was used for rescue embolization in 3 cases due to CPP recurrence 1 month after initial embolization using microcoils (Figure 3). The concentration ratio of Glubran-2 to ethiodized oil was 1:3–1:4, and the mean injected volume was 3.7 ± 1.0 ml (range, 3–5 ml). Final venography revealed complete occlusion of all targeted varicose veins; thus, embolization with Glubran-2 was technically successful for all patients. No Glubran-2-related complications occurred mid-procedure or post-intervention.

Pre-intervention, 3 patients experienced mild pain, 6 patients experienced moderate pain, and 12 patients experienced severe pain. During follow-up (mean 62 ± 38 months; range, 12–102 months), neither persistent nor recurrent CPP requiring repeat endovascular treatment or surgery was observed after embolization with Glubran-2. Clinical efficacy was evaluated at the 1st month [(7.57 ± 1.81 vs. 2.29 ± 0.76), 95% CI 4.257–6.314], 3rd month [(7.57 ± 1. 81 vs. 1.29 ± 0.76), 95% CI 5.257–7.315], 6th month [(7.57 ± 1. 81 vs.0.86 ± 0.69), 95% CI 5.330–8.100], and 12th month [(7.57 ± 1. 81 vs.0.42 ± 0.53), 95% CI 5.789–8.500], and complete or slight improvement of CPP after embolization was observed for all patients. The VAS scores decreased significantly from pre-intervention to post-intervention (*p* < 0.001). The embolization procedural parameters and VAS scores are shown in Table 2. The QOL score at 12 months was 0.19 ± 0.40. Notably, 6 young patients gave birth to healthy babies during the follow-up of Glubran-2 embolization of the ovarian vein.

**Table 2:** Procedural parameters for the proposed treatment and visual analog scale (VAS) scores obtained during follow-up.

**Characteristics**	**Value**
**Time of NBCA Glubran-2 use**, ***n*** **(%)**
First	18 (85.7)
Second	3 (14.3)
**Concentration ratio of NBCA Glubran-2 to ethiodized oil**, ***n*** **(%)**	
1:3	12 (57.1)
1:4	9 (42.9)
NBCA Glubran-2 volume, mL, mean ± SD	3.7 ± 1.0
Number of patients treated using adjuvant microcoils, *n* (%)	4 (19.0)
**Clinical success**, ***n*** **(%)**
Complete	18 (85.7)
Slight	3 (14.3)
**All complications**, ***n*** **(%)**	0 (0)
**VAS scores**
Pretreatment	7.57 ± 1.81
1st month	2.29 ± 0.76
3rd month	1.29 ± 0.76
6th month	0.86 ± 0.69
12th month	0.42 ± 0.53

*NBCA, N-butyl-2 cyanoacrylate; VAS, visual analog scale*.

*Continuous data are presented as the mean ± SD; categorical data are given as counts (percentages)*.

## Discussion

Pelvic Venous Disorder (PeVD) is an underappreciated cause of CPP and disability in young women (3). Living with CPP secondary to PeVD is difficult as this condition directly affects female patients and interactions with family, friends, and general outlook on life. Thus, several treatment modalities for CPP have been proposed over time (3, 6, 17). Conservative treatment involving the use of psychotropic or non-steroidal anti-inflammatory drugs provides only short-term relief from CPP for patients awaiting further investigation or more permanent treatment and plays a minor role in sustained long-term management (18). Hysterectomy is a surgical alternative that fails to reduce CPP symptoms and has therefore been eliminated as a first option (19). Open or laparoscopic surgery to ligate insufficient veins has been proposed as a replacement treatment (13, 20, 21). To date, a consensus regarding indications for either surgery or endovascular embolization of the ovarian veins is lacking, and this topic requires further research. Endovascular embolization treatment has the advantages of minimal invasiveness and has become widely accepted during this century as one of the most effective treatment options available (3, 5–8, 15–17, 19).

Various embolic materials are used during OVE, the most common of which involve liquid sclerosing agents and metal coils, and several retrospective case series have been published on the use of these materials (7, 8, 16, 22). Kim et al. performed a case series using foam-sclerosant embolization, and the VAS significantly decreased from 7.6 ± 1.8 pre-intervention to 2.9 ± 2.8 at a follow-up of 45 ± 18 months. Laborda et al. investigated a group of patients who underwent metal-coil embolization and observed a similar significant reduction in the VAS score from 7.34 ± 0.7 pre-intervention to 0.78 ± 1.2 at a follow-up of 5 years (16, 22). Unfortunately, solid data supporting the superiority of one material over another are lacking. In the present study, a successful outcome was achieved using Glubran-2, and the VAS score decreased from 7.57 ± 1.81 pre-intervention to 0.86 ± 0.69 at the 6-month follow-up. This value corresponds to a similar efficacy but a shorter relief time and an improved QOL was noted at the 1-year follow-up. The results of this study may be attributed to the physicochemical properties of Glubran-2, which functions independently of the hemostatic capacity, such that polymerization can occur immediately upon contact with blood, leading to instant and complete occlusion of insufficient venous axes. In addition, to prevent the risk of embolic material migration, microcoils were used in 4 patients at the opening of the ovarian vein trunk following embolization with Glubran-2, and no migration events occurred in the present study. Note that 3 patients in the present study did not obtain substantial relief from symptoms and experienced CPP recurrence after initial microcoil embolization. This response may have been caused by incomplete occlusion of multiple ovarian vein trunks that occurred in 24–40% of patients (17). Finally, performing embolization using Glubran-2 as a second treatment provided CPP relief. The absence of robust supporting evidence notwithstanding, Glubran-2 appears to have the potential to treat the multiple small tributaries that are often associated with ovarian veins and may cause CPP recurrence.

Safety is an important consideration in the evaluation of OVE. However, complications from this endovascular treatment have rarely been reported in practice (1, 5, 6). One major complication of OVE is non-target vein embolization, which may be caused by using incorrect concentration ratios of glue/coils or protrusions. Another common complication is the migration of coils or glue fragments, which may be attributed to incorrect evaluation of the pelvic vein diameter due to vasospasm (1, 23). Fortunately, these complications were not observed in our study, which could be attributed to the physicochemical properties of Glubran-2 and the knowledge and experience of the clinicians in employing Glubran-2. Moreover, after OVE, no significant changes in the levels of follicle stimulating hormone, luteinizing hormone, or estradiol, which are not associated with pregnancy, were noted (1, 22, 24). Data from a previous study showed that the use of Glubran-2 to treat postpartum hemorrhage did not adversely affect uterine function while embolizing uterine arteries (25). However, there is insufficient evidence to determine whether the reproductive function was affected in our study. However, of note, 6 patients who underwent Glubran-2 embolization of the ovarian vein gave birth to healthy babies.

The coaxial catheter technique was used for all patients in this study. Before microcoils were used, a compatible 2.4-F microcatheter was coaxially positioned to ensure that the microcatheter tip tracked the dilated ovarian vein as closely as possible to enter the target varicose veins of the utero-ovarian plexus. Then, Glubran-2 was injected under withdrawal from the distal insufficient tributary branches into the proximal trunk to achieve precise occlusion of the origin of the leak. The scarcity of adverse events of instantaneous adhesion using the employed microcatheter was attributed to the hydrophilic surface coating of the microcatheter tip. Even in the event of microcatheter adhesion, the microcatheter can be drawn back safely under the protection of a Cobra catheter to prevent colloidal overflow. Note that from practical experience, the use of Glubran-2 appears to be more economical than using microcoils alone.

The present study has several limitations. First, as pain levels are typically subjective, the use of the VAS to evaluate CPP may have introduced a bias into the results, and no strict significant evaluation of QOL scores was performed. Second, considering all the inherent limitations of our relatively small and retrospective study, a multi-institutional prospective study may be required to obtain conclusive results. Third, as the aim of the present study was to evaluate the preliminary outcome of OVE with Glubran-2 for the treatment of CPP secondary to PeVD, the efficacy of Glubran-2 was not compared with conventional embolic materials, which may be necessary. Despite all the above mentioned limitations, to the best of our knowledge, the present study is the largest case series of embolization with Glubran-2 as the sole embolic material for evaluating CPP secondary to PeVD.

In conclusion, OVE with NBCA Glubran-2 is a rapid, feasible, and safe treatment for CPP secondary to PeVD. Notably, the use of the NBCA Glubran-2 appears to be a potential and attractive alternative when patients desire substantial symptom relief and the ability to continue reproducing. Glubran-2 can be used to perform complete embolization of pelvic varicosities through an ovarian vein without the risk of the migration of embolic material. Large studies are warranted to confirm the findings of the present study.

## Data Availability Statement

The original contributions presented in the study are included in the article/supplementary material, further inquiries can be directed to the corresponding authors.

## Ethics Statement

The studies involving human participants were reviewed and approved by the Institutional Review Board (IRB) of the Nanjing First Hospital, Nanjing Medical University (Nanjing, China). The patients/participants provided their written informed consent to participate in this study.

## Author Contributions

MG contributed to data collection and manuscript writing and editing. XH and BZ contributed to project development, data collection, and data analysis. JK and JG contributed to project development, data collection, data analysis, and manuscript editing. HS contributed to project development. All authors contributed to the article and approved the submitted version.

## Conflict of Interest

The authors declare that the research was conducted in the absence of any commercial or financial relationships that could be construed as a potential conflict of interest.

## Publisher's Note

All claims expressed in this article are solely those of the authors and do not necessarily represent those of their affiliated organizations, or those of the publisher, the editors and the reviewers. Any product that may be evaluated in this article, or claim that may be made by its manufacturer, is not guaranteed or endorsed by the publisher.
